# Percutaneous Coronary Intervention in Myocardial Bridging

**DOI:** 10.1016/j.jscai.2022.100563

**Published:** 2022-12-19

**Authors:** Rahul Sawhney, Jared Christensen, Justin Schaffer, Karim Al-Azizi

**Affiliations:** a Cardiology, Baylor Scott & White The Heart Hospital, Plano, Texas; b Medicine, Texas A&M College of Medicine, Dallas, Texas

**Keywords:** dobutamine iFR, intravascular ultrasound, myocardial bridging, percutaneous coronary intervention, physiologic challenge

## Clinical Vignette

**Figure 1:**
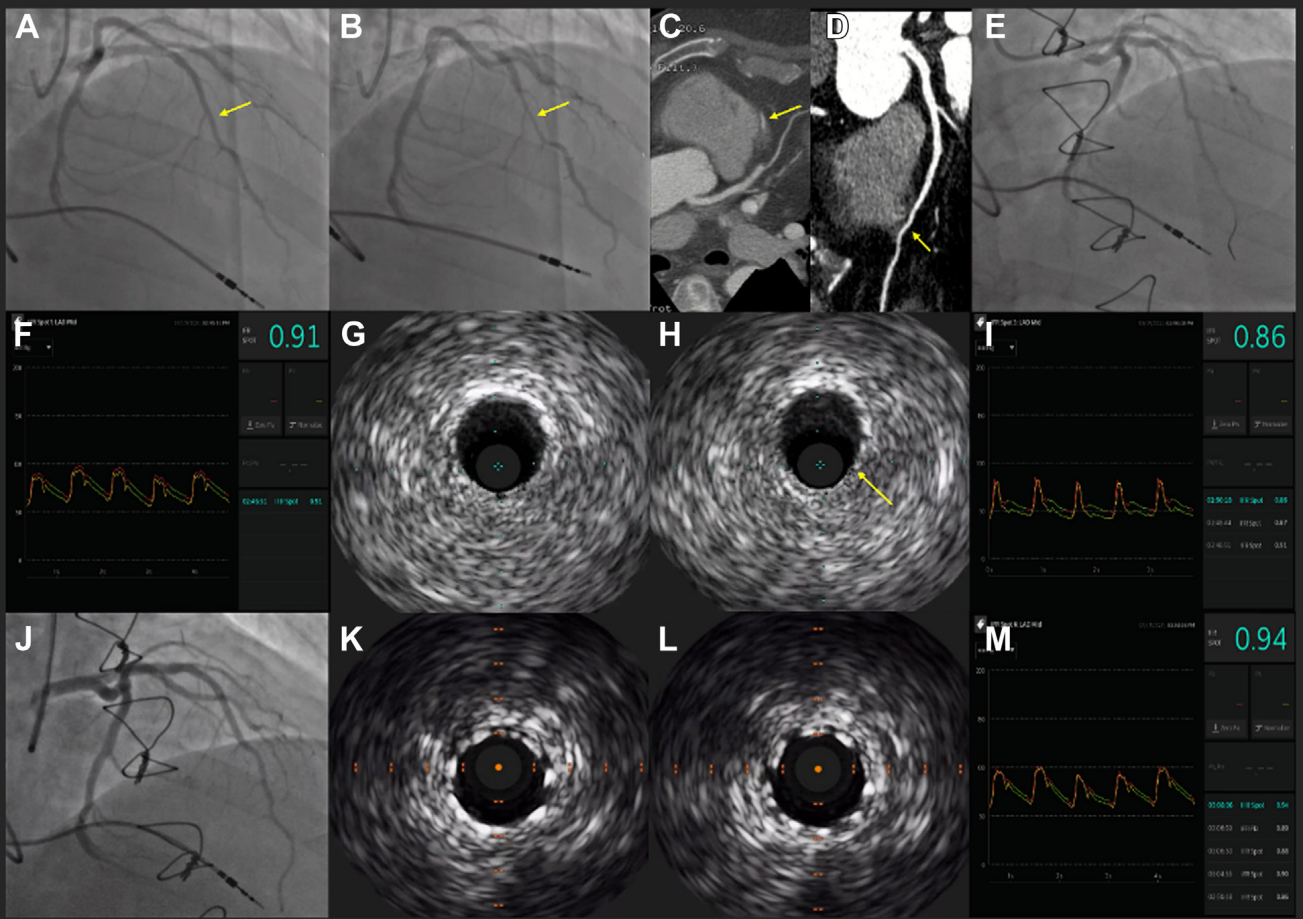
**Multimodality assessment of a myocardial bridging segment.** (**A**, **B**) Coronary angiography of a bridged segment (arrows) in the mid-LAD. (**C**) Coronary CTA showing intramyocardial course (arrow) of the mid LAD (preoperative). (**D**) Coronary CTA showing intramyocardial course (arrow) of the distal LAD (postoperative). (**E**) Coronary angiography of the mid-to-distal LAD without evidence of recurrent bridging. (**F**) iFR of the mid-to-distal LAD showing no ischemia at rest. (**G-H**) IVUS of the mid-to-distal LAD showing dynamic vessel compression (arrow) on 20.0 μg/kg/min of dobutamine. (**I**) iFR of the mid-to-distal LAD showing ischemia on dobutamine. (**J**) PCI of the mid-to-distal LAD with a 2.75 × 26.0-mm Resolute Onyx DES (Medtronic), postdilated to 3.0 mm proximally. (**K-L**) IVUS showing excellent stent apposition, expansion, and lack of dynamic vessel compression on 20.0 μg/kg/min of dobutamine. (**M**) iFR of the mid-to-distal LAD showing no further ischemia while on dobutamine. CTA, computed tomography angiogram; DES, drug-eluting stent; iFR, instantaneous wave-free ratio; IVUS, intravascular ultrasound; LAD, left anterior descending coronary artery; PCI, percutaneous coronary intervention.

A 51-year-old man complained of stable angina. Exercise single-photon emission computed tomography was unremarkable for ischemia. Echocardiogram showed normal biventricular function without hemodynamically significant valvular disease. Because of ongoing symptoms, invasive coronary angiography was performed, which showed severe mid left anterior descending coronary artery (LAD) bridging (Figure 1A, B) with otherwise normal coronary arteries. Despite medical therapy, the patient’s symptoms worsened, and he was referred for surgical unroofing of the LAD. Preoperative coronary computed tomography angiogram (Figure 1C) showed a calcium score of zero, no coronary artery disease, and proximal-to-mid intramyocardial course of the LAD. Intraoperatively, the proximal vessel was clearly epicardial and the mid portion dove underneath 5.0 to 10.0 mm of muscle. The overlying muscle was resected to the proximal takeoff of the D1 (Supplemental Figure). After surgery, the patient’s angina resolved.^1^ Two years later, he experienced recurrence of the same anginal symptoms. Invasive coronary angiography was performed twice and did not demonstrate recurrent bridging. Medical therapy was intensified, but his angina persisted. Repeat coronary computed tomography angiogram (Figure 1D) demonstrated proximity of the mid-to-distal LAD to the myocardium. Pharmacologic positron emission tomography was performed and did not show the presence of ischemia or infarction. Invasive coronary angiography was performed (Figure 1E) with physiologic challenge and intracoronary imaging. Instantaneous wave-free ratio at rest was 0.92 and decreased to 0.84 with dobutamine challenge at 20.0 μg/kg/min (Figure 1F, I).^2^ Intravascular ultrasound performed on dobutamine demonstrated dynamic compression on the ventricular/inferior side of the unroofed LAD (Figure 1G-H). After discussion with the heart team, the consensus was that the LAD had too much flow to support a left internal mammary artery-LAD graft. Therefore, percutaneous coronary intervention (PCI) was performed (Figure 1J) for alleviation of symptoms with the intention of left internal mammary artery-LAD graft in the future if/when in-stent restenosis developed. After PCI, intravascular ultrasound showed excellent stent apposition, expansion, and lack of further coronary artery compression (Figure 1K-L). Moreover, after PCI, dobutamine instantaneous wave-free ratio improved to 0.94 (Figure 1M). The patient remained chest pain free at the 3-month follow-up.

## References

[1] Lee M.S., Chen C. (2015). Myocardial bridging: an up-to-date review. J Invasive Cardiol.

[2] Corban M.T., Hung O.Y., Eshtehardi P. (2014). Myocardial bridging: contemporary understanding of pathophysiology with implications for diagnostic and therapeutic strategies. J Am Coll Cardiol.

