# Supplementary figures and images for: Percutaneous Coronary Intervention in Myocardial Bridging
Source: J Soc Cardiovasc Angiogr Interv. 2022 Dec 19;2(2):100563. doi: 10.1016/j.jscai.2022.100563 (PMC11307909; doi:10.1016/j.jscai.2022.100563)

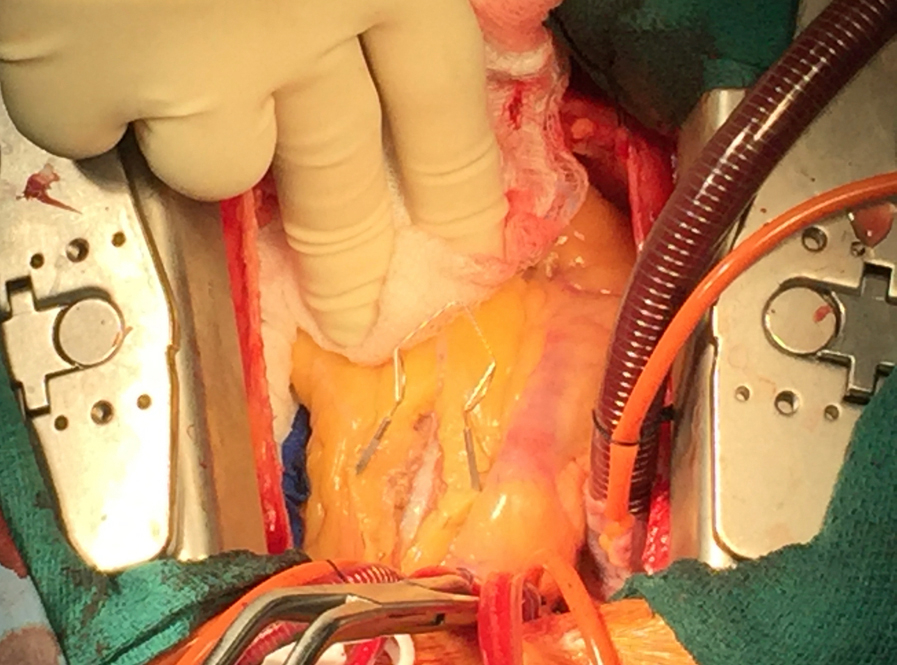

Supplement: supplementary figure [file figs1.jpg]
